# Interactions of human monoclonal and polyclonal antiphospholipid antibodies with serine proteases involved in hemostasis

**DOI:** 10.1002/art.30525

**Published:** 2011-11

**Authors:** Anastasia Lambrianides, Tabitha Turner-Stokes, Charis Pericleous, Jasmine Ehsanullah, Eva Papadimitraki, Katie Poulton, Yiannis Ioannou, Andrew Lawrie, Ian Mackie, Pojen Chen, David Latchman, David Isenberg, Anisur Rahman, Ian Giles

**Affiliations:** 1 University College LondonLondon, UK; 2 University of CaliforniaLos Angeles

## Abstract

**Objective:**

To characterize the interaction between procoagulant and/or anticoagulant serine proteases and human monoclonal IgG antiphospholipid antibodies (aPL) and polyclonal IgG derived from patients with the antiphospholipid syndrome (APS).

**Methods:**

Five human monoclonal IgG with small differences in their sequences were tested for binding to protein C, activated protein C, plasmin, factor VIIa (FVIIa), FIX, FIXa, and FXII. Serum levels of antithrombin and anti–activated protein C were compared in 32 patients with APS, 29 patients with systemic lupus erythematosus (SLE), and 22 healthy controls. Purified polyclonal IgG derived from APS patients with elevated levels of serum antithrombin antibodies was also tested for its functional effects on thrombin and antithrombin activity.

**Results:**

Studies of monoclonal antibodies showed that sequence changes in human aPL are important in determining their ability to bind procoagulant and anticoagulant/fibrinolytic serine proteases. Mean IgG antithrombin levels were significantly elevated in patients with APS and in SLE patients with aPL but no APS (SLE/aPL+) compared to healthy controls, but anti–activated protein C levels were not increased in these patients. Moreover, IgG purified from patients with APS displayed higher avidity for thrombin and significantly inhibited antithrombin inactivation of thrombin compared with IgG from SLE/aPL+ patients.

**Conclusion:**

High-avidity antithrombin antibodies, which prevent antithrombin inactivation of thrombin, distinguish patients with APS from SLE/aPL+ patients, and thus may contribute to the pathogenesis of vascular thrombosis in APS.

Antiphospholipid antibodies (aPL) cause vascular thrombosis and/or pregnancy morbidity in the antiphospholipid syndrome (APS) (1). These clinical manifestations are triggered by the interaction of pathogenic aPL with various target cells, including monocytes, endothelial cells, and trophoblast cells, leading to the recruitment of cell surface receptors and subsequent perturbation of intracellular signaling pathways (2). These pathogenic aPL are generally IgG type (3, 4) and target a variety of antigens, including negative phospholipid, phospholipid binding proteins (particularly β_2_-glycoprotein I [β_2_GPI] and prothrombin), as well as other factors related to hemostasis, such as thrombin, protein C, activated protein C, protein S, plasmin, plasminogen, and tissue-type plasminogen activator (tPA) (5–13). In contrast, nonpathogenic aPL (found in 2–5% of healthy adults who lack features of the APS [14]) mostly bind directly to phospholipid (15).

Thrombin, activated protein C, plasmin, and tPA, as well as activated factor VIIa (FVIIa), FIXa, FXa, and FXIIa, belong to the trypsin-like serine protease family of enzymes and are involved in the tight regulation of hemostasis (16). In previous studies, sera from between 13% and 54% of patients with the APS have been found to bind various different serine proteases (5, 8, 13). Furthermore, a panel of human monoclonal aPL produced from hybridomas displayed cross-reactivity with serine protease, binding to thrombin, activated protein C, plasmin, tPA, FIXa, and FXa (6–8, 17, 18). Overall, these serine proteases share ∼50% amino acid sequence similarity in their enzymatic domains but have greater homology at their catalytic sites. Given that several human monoclonal aPL have been found to inhibit the inactivation of procoagulant serine proteases and functional activities of anticoagulant/fibrinolytic serine proteases (7, 8, 13, 19), it has been suggested that some aPL may recognize the catalytic domain of serine proteases, leading to dysregulation of hemostasis and vascular thrombosis in the APS.

To explore the interaction of aPL with target antigens in promoting thrombus formation, we have been studying a panel of recombinant human monoclonal IgG aPL, which differ from one another at points in their sequence precisely engineered by us. Studying this panel of IgG molecules has allowed us to investigate correlations between their sequences, binding, and biologic properties (20–23). These human monoclonal IgG aPL were all based on the human monoclonal IgG aPL IS4 (derived from a patient with APS), which binds β_2_GPI (24) and thrombin (8) and is thrombogenic in mice (25). Previously, we found that alterations in the pattern of somatic mutations in both the V_H_ and V_L_ regions of IS4 determined its ability to bind antigens relevant in the pathogenesis of the APS and to promote murine thrombogenesis (20–23). Interestingly, the in vivo thrombogenic effects of these monoclonal antibodies (mAb) were most closely predicted by their ability to bind thrombin, rather than phospholipid or β_2_GPI. Furthermore, mAb binding to thrombin followed a different pattern compared to the pattern observed with mAb binding to its zymogen prothrombin (21).

Therefore, in the current study we used the same panel of mAb to examine whether binding to other serine proteases also parallels thrombogenicity in the mouse model, and whether the difference between binding to prothrombin and binding to thrombin is also seen with other zymogen/enzyme pairs, i.e., FIX and FIXa, or protein C and activated protein C. To assess the relevance of our findings obtained using monoclonal IgG aPL to polyclonal aPL found in vivo, we then tested serum samples and purified IgG samples from APS patients, systemic lupus erythematosus (SLE) patients without APS (subclassified according to positivity or negativity for aPL), and healthy controls. We investigated whether samples from those groups differed in the nature and avidity of their binding to serine proteases and ability to alter the functional activity of serine proteases.

## PATIENTS AND METHODS

### Human monoclonal IgG antibodies

Production of the antibodies (IS4VH/IS4VL, IS4VH/B3VL, IS4VH/UK4VL, IS4VHi&ii/IS4VL, and IS4VHi&ii/B3VL) has been well described (21, 23, 26, 27). Variant forms of IgG were produced by site-directed mutagenesis in IS4VHCDR3 and/or by replacing IS4VL with similar V_L_ chains from an antinucleosome mAb (B3 [28]) or a β_2_GPI-independent aPL (UK4 [29]). These V_L_ chains were all derived from the germline V_λ_ gene (2a2), sharing at least 93% sequence homology and differing solely in their pattern of somatic mutation (23). IS4VHi&ii differs from IS4VH in 2 arginine-to-serine mutations at positions 96 and 97. An irrelevant nonbinding monoclonal IgG antibody was produced in an identical manner and used as a negative control. Large-scale production and purification of IgG was performed by an outside company (Harlan). The concentration of IgG was confirmed by both total IgG enzyme-linked immunosorbent assay (ELISA) (23) and spectrophotometry.

### Patients and healthy controls

Serum samples for this study were obtained from 83 individuals (patients under our care at University College London Hospital and healthy controls) (Table 1). All subjects had provided written informed consent. Of 32 patients fulfilling the revised classification criteria for APS (1), 14 also had SLE fulfilling the American College of Rheumatology (ACR) classification criteria (30) and 18 had primary APS. Consistent with findings from other cohort studies (31, 32) the APS-related clinical and serologic features in our primary APS and SLE/APS groups were similar, and these patients were therefore combined into one group called APS. As an autoimmune disease control group we obtained samples from 29 patients who had SLE (fulfilling the ACR criteria) but did not have APS. Thirteen were aPL positive (SLE/aPL+) and 16 were aPL negative (SLE/aPL−). The healthy control group consisted of 22 individuals. To ensure that any residual thrombin present in serum was rapidly inhibited by antithrombin or α_2_-macroglobulin and/or absorbed by fibrin, all patient/control blood samples were left to clot for 2 hours before centrifugation and collection of serum. Results of experiments to confirm that there was no residual thrombin activity in serum at dilutions used in subsequent ELISAs are available at http://discovery.ucl.ac.uk/1316886/.

**Table 1:** Clinical and laboratory features of the subjects studied*

	APS (n = 22)	SLE/aPL+ (n = 13)	SLE/aPL− (n = 16)	Healthy controls (n = 22)
Age, mean years	49.59	41.69	39.44	35.32
Sex, male/female	0/32	0/13	2/14	9/13
Vascular thrombosis	DVT (n = 13), PE (n = 9), CVA (n = 9), TIA (n = 5)	None	DVT (n = 1), PE (n = 1)	None
Pregnancy morbidity	RM (n = 31), FD (n = 17)	None	FD (n = 1)	None
Other ARD	SLE (n = 14)	None	None	None
Treatment	Aspirin (n = 17), warfarin (n = 12), steroids (n = 5), immunosuppressive drugs (n = 9)	Aspirin (n = 9), warfarin (n = 1), steroids (n = 9), immunosuppressive drugs (n = 8)	Aspirin (n = 4), steroids (n = 12), immunosuppressive drugs (n = 13)	None
aCL, mean GPL units	55.28	8.61	13.36	11.82
Anti-β_2_GPI, mean AU	22.34	19	0.25	0.26
LAC positive	23	9	0	0

* Except where indicated otherwise, values are the number of patients. APS = antiphospholipid syndrome; SLE/aPL+ = antiphospholipid antibody–positive systemic lupus erythematosus; SLE/aPL− = antiphospholipid antibody–negative systemic lupus erythematosus; DVT = deep vein thrombosis; PE = pulmonary embolism; CVA = cerebrovascular accident; TIA = transient ischemic attack; RM = recurrent miscarriages (≥3 first-trimester miscarriages); FD = fetal death; ARD = autoimmune rheumatic disease; aCL = anticardiolipin antibody; GPL = IgG phospholipid; anti-β_2_GPI = anti–β_2_-glycoprotein I; AU = arbitrary units; LAC = lupus anticoagulant

### Purification and immunologic characterization of IgG

All IgG was purified by protein G–Sepharose affinity chromatography (GE Healthcare Lifesciences). The concentration of purified IgG was determined using a Nanodrop ND-1000 Spectrophotometer (LabTech International). Anticardiolipin antibody (aCL) and IgG anti-β_2_GPI titers were measured in all serum samples as previously described (21), using international calibrators (Louisville APL Diagnostics) and the IgG Sapporo standard, HCAL (Centers for Disease Control and Prevention) (1). Antithrombin antibodies were detected as described previously (8, 20).

### Antiplasmin ELISA

IgG antiplasmin antibodies were detected using the method described by Yang et al (13). The test half of a high-binding Costar plate was coated with 5 μg/ml human plasmin (Haematologic Technologies) in phosphate buffered saline (PBS); PBS alone was used on the control half. Plates were incubated overnight at 4°C and blocked with PBS/0.25% gelatin for 1 hour at room temperature. Monoclonal IgG (100 μg/ml) in PBS/0.1% gelatin was incubated for 1.5 hours at room temperature. Bound IgG was detected by addition of anti-human IgG Fc–specific alkaline phosphatase conjugate in PBS/0.1% gelatin for 1 hour followed by addition of substrate, and absorbance was read at 405 nm.

### Anti–factor VIIa ELISA

IgG anti-FVIIa antibodies were detected according to the method described by Bidot et al (33). MaxiSorp plates were coated with 1.5 μg/ml recombinant human FVIIa (Novo Nordisk) in PBS on the test half and PBS alone on the control half. Plates were incubated overnight at 4°C and then blocked with 200 μl PBS/0.1% Tween/2% bovine serum albumin (BSA) for 2 hours at room temperature. Monoclonal IgG (100 μg/ml) in PBS/1% BSA was incubated at room temperature for 1 hour. Bound IgG was detected by addition of anti-human IgG Fc–specific alkaline phosphatase conjugate in PBS/1% BSA for 1 hour followed by addition of substrate, and absorbance was read at 405 nm.

### Anti–factor XII ELISA

To detect IgG anti-FXII antibodies, a modification of the method of Jones et al (34) was used. MaxiSorp plates were coated with 5 μg/ml FXII (Haematologic Technologies) in carbonate–bicarbonate buffer on the test half of the plate and carbonate–bicarbonate buffer alone on the control half. Plates were then incubated for 1 hour at room temperature and blocked with Tris buffered saline (TBS)/2% BSA for 1 hour at room temperature. Monoclonal IgG (100 μg/ml) in TBS/1% BSA was incubated at room temperature for 1 hour and bound IgG detected by the addition of anti-human IgG Fc–specific alkaline phosphatase for 1 hour followed by addition of substrate, and absorbance was read at 405 nm.

### Anti–protein C and anti–activated protein C ELISA

Anti–protein C and anti–activated protein C binding was measured as described by Hwang et al (19). The test half of a high-binding Costar plate was coated with 5 μg/ml protein C or activated protein C (Haematologic Technologies) in TBS/2.5 m*M* CaCl_2_; TBS/2.5 m*M* CaCl_2_ alone was used on the control half. The plates were washed with TBS/2.5 m*M* CaCl_2_ and blocked using TBS/2.5 m*M* CaCl_2_/0.3% gelatin. Monoclonal IgG was diluted in TBS/2.5 m*M* CaCl_2_/0.1% gelatin and incubated for 1 hour at room temperature. For testing of serum the assay was modified, with protein C/activated protein C used at 10 μg/ml, serum diluted 1:25 in TBS/2.5 m*M* CaCl_2_/0.1% gelatin, and incubation carried out for 1.5 hours.

### Anti–factor IX and anti–factor IXa ELISA

Anti-FIX and anti-FIXa antibodies were detected using the method of Yang et al (17). The test half of a high-binding Costar plate was coated with 5 μg/ml FIX or FIXa (Haematologic Technologies) in TBS; TBS alone was used on the control half. Plates were incubated overnight at 4°C and blocked with 100 μl TBS/0.3% gelatin for 1 hour at room temperature. Monoclonal IgG (100 μg/ml) in TBS/0.1% gelatin was incubated at room temperature for 1.5 hours. Bound IgG was detected by addition of anti-human IgG Fc–specific alkaline phosphatase conjugate in TBS/0.1% gelatin for 1 hour followed by addition of substrate, and absorbance was read at 405 nm.

### Chaotropic ELISA for determination of avidity of antithrombin antibodies

A chaotropic ELISA for antithrombin antibody avidity was adapted from that described by Cucnik et al (35), whose chaotropic ELISA was established using NaCl to measure the avidity of IgG–β_2_GPI interactions in patients with APS. Briefly, high-binding Costar plates were coated with 10 μg/ml human α-thrombin, incubated overnight, and blocked as described above. IgG was purified from the serum of patients who were positive for antithrombin antibodies (absorbance units [AU] more than 3 SD above the mean in the control group). Purified IgG (200 μg/ml) in TBS/0.1% gelatin containing increasing concentrations of NaCl (0.15*M* [Tris buffer alone], 0.25*M*, 0.35*M*, 0.5*M*, 1*M*, 2*M*, 3*M*, and 4.5*M*) was loaded onto the plate and incubated for 1.5 hours at room temperature, and bound IgG detected as described above. Avidity was determined by calculating the percentage of maximum binding (at 0.15*M* NaCl) maintained with each concentration of NaCl and comparing this between samples.

### Functional assay for thrombin activity and antithrombin inactivation of thrombin

The effects of thrombin-reactive IgG on thrombin activity were studied as previously described (8), with minor modifications. Briefly, 80 n*M* human α-thrombin (Hyphen Biomed) was mixed with IgG (final concentration 100 μg/ml) and incubated for 1 hour at room temperature. Subsequently, 150 μ*M* of the thrombin chromogenic substrate S-2238 (Chromogenix) was added, and after 2 minutes, generation of *P*-nitroaniline was monitored by measuring optical density (OD) at 405 nm. The activity of thrombin was determined based on the rate of hydrolysis of S-2238 from the linear range of absorbance at 405 nm over time.

The effects of thrombin-reactive IgG on thrombin inactivation by antithrombin were studied as described by Bock et al (36), with minor modifications. Briefly, 6.7 n*M* of thrombin was incubated with IgG (final concentration 100 μg/ml) in a HEPES/NaCl/EDTA/0.1% polyethylene glycol (pH 7.4) buffer for 1 hour at room temperature. Then, 67 n*M* of antithrombin in the same buffer, but also containing heparin (0.1 IU/ml), was added, followed immediately by addition of S-2238, and OD at 405 nm was measured (at 2 minutes, unless otherwise stated). The percentage of thrombin inactivation by antithrombin was calculated as [1 − (residual thrombin activity with antithrombin)/(initial thrombin activity without antithrombin)] × 100.

### Statistical analysis

Data analysis was performed using GraphPad Prism software. Normality of distribution was assessed using the Kolmogorov-Smirnov test. The effects of monoclonal IgG on FVIIa, FIXa, FIX, FXII, plasmin, activated protein C, and protein C were compared by Kruskal-Wallis test with one-way analysis of variance (ANOVA) followed by Dunn's post hoc test. Differences in antithrombin and anti–activated protein C antibody titers between patient groups were compared by one-way ANOVA with post hoc analysis by Bonferroni test. The association of antithrombin antibody titers with aCL, anti-β_2_GPI, and anti–activated protein C antibody titers was assessed using Spearman's rank correlation coefficient. The significance of differences in the avidity of antithrombin antibodies between APS and SLE patient groups was ascertained by 2-tailed *t*-test. The effects of polyclonal antithrombin IgG from patients with APS, patients with SLE, and healthy controls on antithrombin-mediated inactivation of thrombin were compared using Kruskal-Wallis test followed by Dunn's post hoc test.

## RESULTS

### Binding properties and functional effects of monoclonal IgG on serine proteases

**Figure 1:**
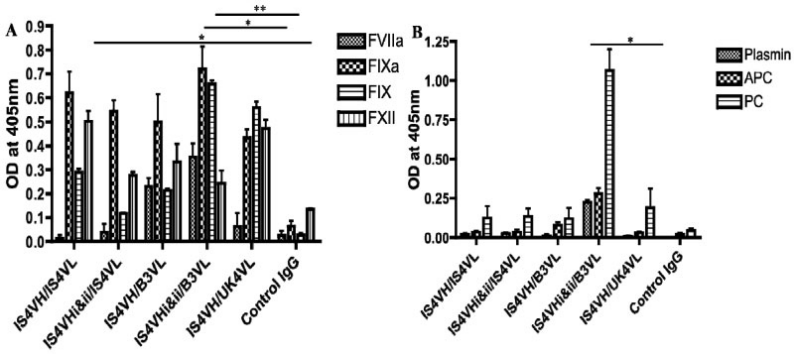
Binding of IS4 variant monoclonal antibodies to procoagulant serine proteases and zymogens and to anticoagulant and fibrinolytic serine proteases and zymogens. A, Binding of each V_H_/V_L_ combination to human factor VIIa (FVIIa), FIXa, FIX, and FXII. B, Binding of each V_H_/V_L_ combination to human plasmin, activated protein C (APC), and protein C (PC). Values are the mean ± SEM optical density (OD) of IgG (100 μg/ml), tested in triplicate. *= *P* < 0.05 versus control IgG (for binding of IS4VH/IS4VL to FXII and binding of IS4VHi&ii/B3VL to FIXa [A] and for binding of IS4VHi&ii/B3VL to plasmin [B]); **= *P* < 0.01 versus control IgG (for binding of IS4VHi&ii/B3VL to FIX [A]).

We examined binding of the monoclonal IgG to 4 procoagulant (FVIIa, FIXa, FIX, and FXII) and 3 anticoagulant/fibrinolytic (plasmin, activated protein C, and protein C) serine protease/zymogens. None of these serine proteases showed the same pattern of binding to these 5 mAb, as previously seen with thrombin (20). Only 2 mAb, one of which had strong antithrombin binding (IS4VHi&ii/B3VL) and the other of which had no antithrombin binding (IS4VH/B3VL), displayed weak binding to FVIIa, which failed to reach statistical significance compared with control IgG (Table 2 and Figure 1A). The other 3 IS4 variants, including native IS4VH/IS4VL itself, exhibited negligible anti-FVIIa binding. In contrast, all of the IS4 variants displayed moderate binding to FIXa and FXII (Table 2 and Figure 1A). Binding of IS4VHi&ii/B3VL to FIXa and binding of IS4VH/IS4VL to FXII were significantly increased (*P* < 0.05) compared with control IgG. Only 2 of the 5 IS4 variants (IS4VHi&ii/B3VL and IS4VH/UK4VL) showed moderate binding to FIX (Table 2 and Figure 1A), with the increase being significant only for IS4VHi&ii/B3VL (*P* < 0.01). Only 1 heavy/light chain combination (IS4VHi&ii/B3VL) displayed any evidence of binding to the fibrinolytic serine protease plasmin (*P* < 0.05 compared with control IgG), the anticoagulant serine protease activated protein C, and the zymogen protein C (Table 2 and Figure 1B).

**Table 2:** Summary of binding and functional characteristics of the 5 heavy/light chain combinations*

Heavy chain/light chain	CL	Thrombin	Plasmin	Protein C	Activated protein C	FVIIa	FIX	FIXa	FXII	Inhibition of thrombin/activated proteinC/antithrombin activity
IS4VH/IS4VL	++	++	−	−	−	−	+	++	++	−
IS4VHi&ii/IS4VL	−	+	−	+	−	−	+	++	+	−
IS4VH/B3VL	++	+	−	−	−	+	+	++	+	−
IS4VHi&ii/B3VL	+++	+++	+	+++	+	+	++	++	+	−
IS4VH/UK4VL	++	+	−	+	−	−	++	++	+	−

* Binding of purified IgG to cardiolipin (CL), thrombin, plasmin, protein C, activated protein C, factor VIIa (FVIIa), FIX, FIXa, and FXII and ability to inhibit thrombin, activated protein C, and antithrombin activity are shown. The identity of native heavy and light chains is clearly indicated. IS4VHi&ii contains 2 Arg-to-Ser replacements at positions 96 and 97. Each V_H_/V_L_ combination was tested at 100 μg/ml in triplicate, and the degree of binding was defined from the mean absorbance, as follows: − = optical density (OD) <0.1; + = OD 0.1–0.4; ++ = OD >0.4–0.8; +++ = OD >0.8–1.2; ++++ = OD >1.2.

We compared binding of monoclonal IgG to 2 zymogen/serine protease pairs. The zymogen protein C and its serine protease activated protein C exhibited very similar patterns of binding (Figure 1B). Only IS4VHi&ii/B3VL displayed greater binding to protein C than to activated protein C.

### Detection of antithrombin and anti–activated protein C binding in the serum of patients with APS

**Figure 2:**
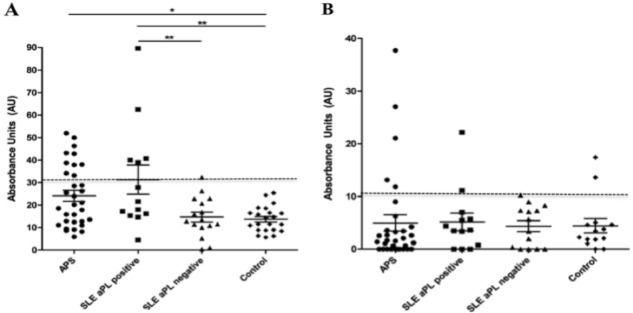
Detection of antithrombin and anti–activated protein C antibodies in serum. Serum from antiphospholipid syndrome (APS) patients, from antiphospholipid antibody (aPL)–positive systemic lupus erythematosus (SLE) patients without APS, from aPL-negative SLE patients without APS, and from healthy controls was tested for the presence of IgG antibodies to thrombin (A) and activated protein C (B). Serum was tested in triplicate at 1:25 dilution, and binding was expressed in arbitrary units (AU) in comparison to the binding of monoclonal antibody IS4VHi&ii/B3VL. Symbols represent individual subjects; bars show the mean ± SD. Dashed lines depict the cutoff for positivity, defined as values more than 3 SD above the mean in healthy controls (n = 22). *= *P* < 0.05; **= *P* < 0.01.

We then examined IgG antithrombin and anti–activated protein C antibodies in patients with APS, patients with SLE, and healthy controls. Figure 2A shows that mean IgG antithrombin levels were significantly increased in the 2 groups of patients who were positive for serum aPL (24.1 AU in the APS group and 31.3 AU in the SLE/aPL+ group, compared with 14.6 AU in the SLE/aPL− group and 13.6 AU in the healthy controls). There were statistically significant differences between the APS group and the healthy controls (*P* < 0.05), between the SLE/aPL+ group and the healthy controls (*P* < 0.01), and between the SLE/aPL+ group and the SLE/aPL− group (*P* < 0.01). The upper limit of normal in this assay was defined as 3 SD above the mean in the healthy control group, i.e., 30.7 AU. Using this cutoff, antithrombin antibodies were present in 10 of the patients in the APS group and 5 of the patients in the SLE/aPL+ group (38.5%), compared to only 1 patient in the SLE/aPL− group (6%).

In contrast, there were no significant differences in IgG anti–activated protein C levels between any of the groups studied (Figure 2B). Anti–activated protein C antibodies were present (i.e., levels more than 3 SD above the mean in healthy controls) in 5 (15.6%) of the APS patients and 2 (15.4%) of the SLE/aPL+ patients. Furthermore, in the 45 subjects who were aPL positive (32 APS and 13 SLE/aPL+), antithrombin titers were not correlated with titers of aCL (r = 0.018, *P* = 0.92), anti-β_2_GPI (r = 0.15, *P* = 0.31), or anti–activated protein C (r = 0.27, *P* = 0.31).

### Avidity of antithrombin antibodies

**Figure 3:**
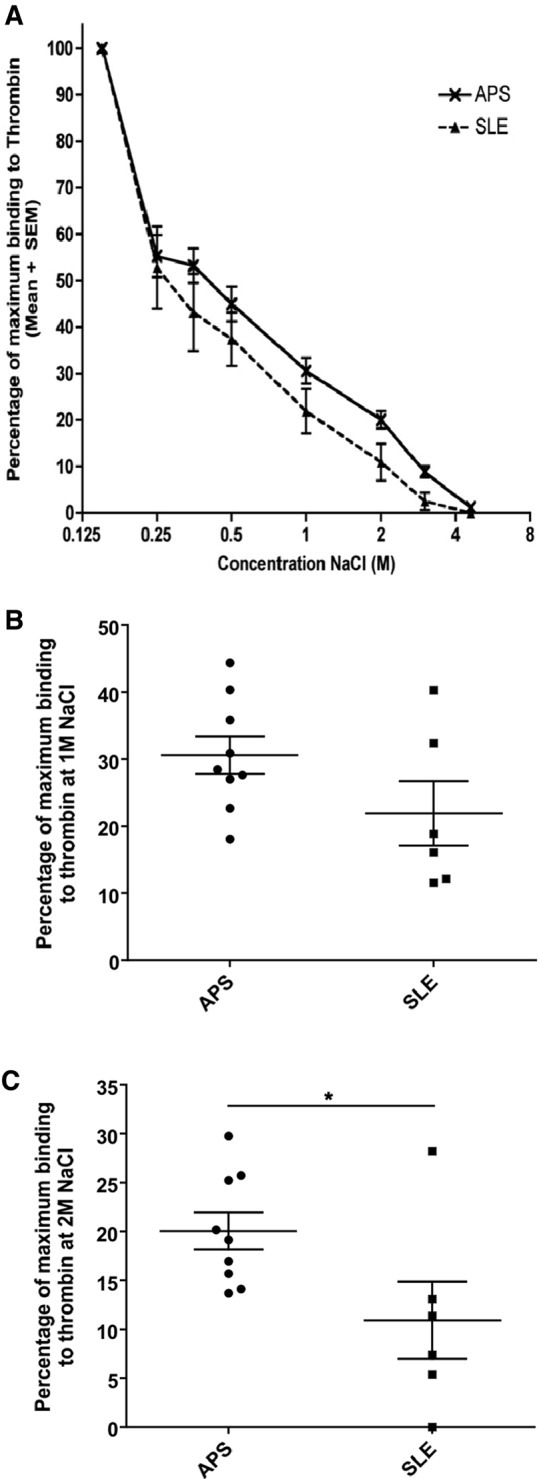
Avidity of antithrombin antibodies from antiphospholipid syndrome (APS) patients compared to systemic lupus erythematosus (SLE) patients with antiphospholipid antibody. A, Percentage of maximum binding to thrombin with NaCl at all concentrations tested. B and C, Percentage of maximum binding to thrombin with NaCl at 1*M* (B) and 2*M* (C). Symbols in B and C represent individual subjects; bars show the mean ± SEM. *= *P* < 0.05.

As described above, 16 patients were found to be positive for antithrombin antibodies (10 with APS, 5 with aPL+ SLE, and 1 with aPL− SLE). To investigate whether there was any difference in the avidity of these antibodies for thrombin between the APS and the SLE/aPL+ groups, we introduced chaotropic conditions to the thrombin ELISA. Interestingly, as the concentration of NaCl was increased above 1*M*, the mean residual binding of polyclonal IgG to thrombin was higher in samples from patients with APS compared to patients with SLE and aPL but without APS (Figure 3A). The difference between the 2 groups increased as the concentration of NaCl increased (Figures 3B and C), reaching statistical significance at 2*M* NaCl (mean residual binding 20.0% in the APS group versus 10.9% in the SLE group; *P* < 0.05) (Figure 3C).

### Functional properties of antithrombin antibodies

**Figure 4:**
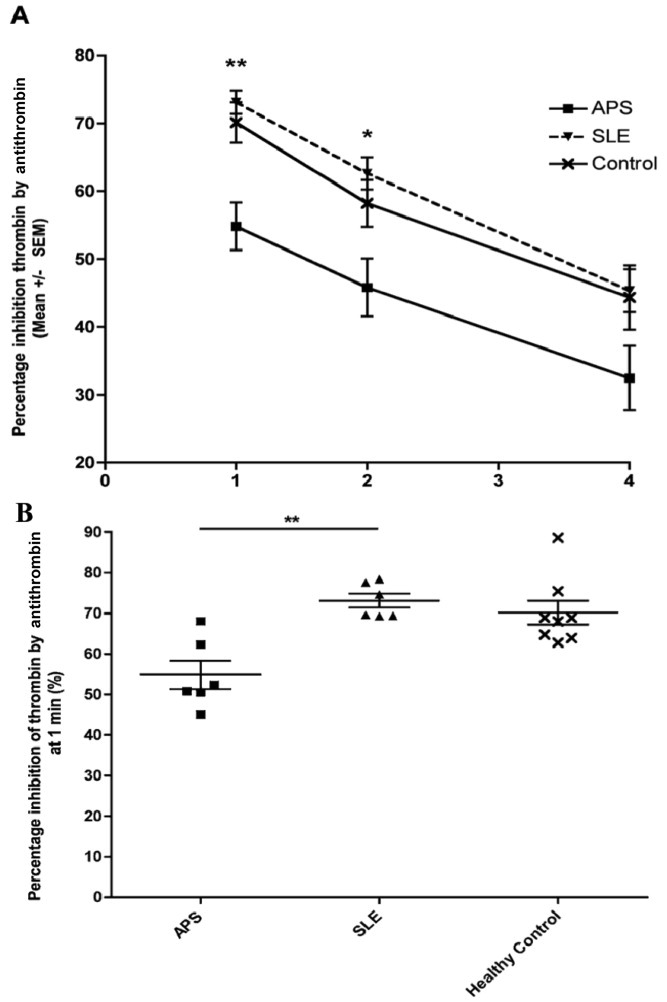
Effect of purified polyclonal IgG from patients with antiphospholipid syndrome (APS) or systemic lupus erythematosus (SLE) on antithrombin-mediated inhibition of thrombin. Purified IgG from 9 of the 10 patients with APS and 6 patients with SLE whose sera contained antithrombin antibodies, as well as from 11 healthy controls, was tested for its ability to prevent antithrombin-mediated inhibition of thrombin. A, Percentage inhibition of thrombin at all time points tested. Values are the mean ± SEM. *= *P* < 0.05; **= *P* < 0.01, SLE patients versus APS patients. No statistically significant differences were observed at time points past 2 minutes. B, Percentage inhibition of thrombin at 1 minute. Symbols represent individual subjects; bars show the mean ± SEM.

To investigate the functional significance of the thrombin-reactive IgG, we examined the effect of IgG purified from the serum of 9 of the 10 antithrombin antibody–positive APS patients on the inhibition of thrombin by antithrombin. We compared the results to those obtained using IgG purified from the sera of the 6 antithrombin antibody–positive patients with SLE but no APS and 7 healthy controls (Figures 4A and B). IgG from patients with APS significantly reduced the inactivation of thrombin by antithrombin compared to IgG isolated from patients with SLE at both 1 minute (*P* < 0.01) (Figure 4B) and 2 minutes (*P* < 0.05) (data not shown), but there was no significant difference between the results obtained using IgG from APS patients and healthy controls. No statistically significant differences were found at 4 minutes; beyond this time point, the linear rate of absorbance plateaus and it is difficult to accurately measure degree of inhibition by antithrombin. When this assay was carried out in the absence of antithrombin, i.e., to determine whether there was any direct effect of the IgG on the action of thrombin, none of the IgG tested had any effect on the activity of thrombin alone (data not shown).

## DISCUSSION

In our previous studies using a panel of 5 human monoclonal IgG aPL (20), only 2 (IS4VH/IS4VL and IS4VHi&ii/B3VL) showed an association between thrombin binding in vitro and ability to promote murine thrombosis in vivo. In the present investigation we demonstrated that this finding is not a class effect common to the 4 other serine proteases and 3 zymogens tested, since binding to these antigens did not distinguish pathogenic (IS4VH/IS4VL and IS4VHi&ii/B3VL) from nonpathogenic (IS4VHi&ii/IS4VL, IS4VH/B3VL, and IS4VH/UK4VL) mAb in the way previously demonstrated for antithrombin binding. Although the combination IS4VHi&ii/B3VL is able to bind all 5 active serine proteases tested so far (thrombin, plasmin, FIXa, FVIIa, and activated protein C), it also binds well to the zymogens FIX, FXII, and protein C, in which the serine protease catalytic site is not exposed. The other thrombogenic combination, IS4VH/IS4VL, also does not bind serine proteases better than zymogens; it binds the serine proteases thrombin and FIXa, but not activated protein C, plasmin, or FVIIa. Hence, only binding to thrombin by a large panel of well-characterized human-derived mAb predicted their pathogenicity in mice. Therefore, we characterized the interaction between human-derived polyclonal antibodies from the sera of patients (APS and control patients) and thrombin, to elucidate the relevance of these findings in disease.

Several different groups have identified anti–serine protease antibodies in patients with APS (5, 7, 8, 13, 17, 18, 33, 37), although the clinical significance of these findings has yet to be established. Since some serine proteases exert procoagulant effects whereas others exert anticoagulant effects, it may prove difficult to identify the effects of anti–serine protease antibodies on hemostasis in vivo. Given our demonstration, in experiments using human monoclonal IgG aPL, of the importance of binding to thrombin, we investigated the nature, avidity, and functional effects of IgG antithrombin antibodies in patients with APS. We found antithrombin antibody levels to be elevated above the cutoff in 31.3% of our APS patient cohort, with no significant correlation between antithrombin antibody levels and aCL or anti-β_2_GPI antibody levels. Direct binding of β_2_GPI to thrombin was recently demonstrated (38), and this binding was subsequently shown to protect against thrombin inactivation by heparin cofactor II, with the procoagulant effect potentiated by anti-β_2_GPI antibodies (39). Our present findings, however, demonstrate that antithrombin binding is not simply a surrogate marker for anti-β_2_GPI binding and that these antibodies are distinct from other aPL. IgG from patients with APS did not inhibit thrombin activity, although these IgG reduced the antithrombin inhibition of thrombin. This suggests that the IgG from patients with APS bind to the exosite or heparin binding site on thrombin, rather than the catalytic site.

The antithrombin antibodies were not specific to APS: 38.5% of the patients with SLE who were positive for aPL but lacked clinical features of APS were also found to have significantly elevated levels of antithrombin antibodies. Although based on samples from a relatively small number of patients, our results (Figures 3 and 4) demonstrate that there are differences between the antithrombin antibodies found in patients with APS and those found in patients with SLE but without APS. The antithrombin antibodies from patients with APS have a higher avidity for thrombin than those from patients with SLE without APS, and the antithrombin-mediated inactivation of thrombin by purified IgG from patients with APS was significantly reduced at time points up to 2 minutes compared with that by purified IgG from patients with SLE.

These findings are relevant to the pathogenesis of APS, since high-avidity antithrombin antibodies, which prevent thrombin inactivation, are more likely to promote vascular thrombosis than are low-avidity antithrombin antibodies, which lack this function. Indeed, previous testing of a panel of hybridoma-derived monoclonal aPL showed that an aPL (named CL24) with the greatest avidity for binding to thrombin exerted the strongest inhibition of antithrombin activity (8) and was thrombogenic in mice (25). These results mirror those of other groups who have demonstrated that high-avidity serum anti-β_2_GPI antibodies are more closely associated with thrombosis than are low-avidity serum anti-β_2_GPI antibodies in patients with APS (35). Consequently, these differences in binding avidity may contribute to the phenotypic differences between APS patients and SLE patients with respect to their predisposition to thrombus formation.

Interestingly, although anti–activated protein C antibodies have been described in patients with the APS, we did not find significantly increased levels of these antibodies in our cohort of APS patients compared to healthy controls (Figure 2B). Hence, despite the fact that the catalytic sites of activated protein C and thrombin share ∼50% amino acid sequence homology, antithrombin antibodies in our patient cohort do not appear to cross-react with activated protein C. Therefore, the results of our experiments on patient serum are consistent with the impression derived from the mAb experiments, i.e., that anti–serine protease antibodies in patients with APS do not interact with epitopes in the shared catalytic sites of serine protease, and that antibodies against the procoagulant serine protease thrombin, rather than the anticoagulant serine protease activated protein C, are associated with promotion of thrombosis.

The end point of coagulation, however, is a series of interactions between inhibitors and procoagulants, leading to thrombin generation. To more thoroughly understand the impact of aPL on the net effect of the coagulation cascade leading to thrombin generation, it is necessary to ascertain their effects in a global coagulation assay measuring endogenous thrombin potential. To begin to address this we have performed preliminary experiments examining the effects of selected IgG on thrombin generation, assessed based on endogenous thrombin potential, under various experimental conditions. We compared 2 IgG samples with high-avidity thrombin binding (from patients with APS) and 2 samples with low-avidity thrombin binding (from patients with SLE) and found no appreciable difference in the effect of these IgG samples on endogenous thrombin potential (data available at http://discovery.ucl.ac.uk/1316886/). Further experiments using activity assays are now needed to investigate the effect of these antibodies on different functions of thrombin, in order to better understand their role in the pathogenesis of the APS.

Our study has some limitations. For pragmatic reasons we were able to analyze binding of serum to only 2 serine proteases: thrombin and activated protein C. It remains possible that antibodies to other procoagulant or anticoagulant serine proteases were important in the pathogenesis of APS in our patients. The chaotropic method that we used to assess avidity of binding yielded interesting results, but antigen–antibody binding under high-salt conditions may also be affected by changes in hydrophobicity of the interaction. In future studies it would be useful to add other methods for measuring avidity, such as surface plasmon resonance.

In conclusion, we have demonstrated that sequence changes in both the V_H_ and the V_L_ regions of human aPL alter their ability to bind procoagulant and anticoagulant/fibrinolytic serine proteases but have no effect on in vitro serine protease activity. Furthermore, we have shown that antithrombin antibodies in patients with APS have high avidity and prevent antithrombin inactivation of thrombin compared to those in aPL-positive patients with SLE but without APS. These properties may contribute to the pathogenesis of vascular thrombosis in APS.

## AUTHOR CONTRIBUTIONS

All authors were involved in drafting the article or revising it critically for important intellectual content, and all authors approved the final version to be published. Dr. Lambrianides had full access to all of the data in the study and takes responsibility for the integrity of the data and the accuracy of the data analysis.

**Study conception and design.** Lambrianides, Pericleous, Ioannou, Lawrie, Mackie, Latchman, Isenberg, Rahman, Giles.

**Acquisition of data.** Lambrianides, Turner-Stokes, Pericleous, Ehsanullah, Papadimitraki, Poulton, Lawrie.

**Analysis and interpretation of data.** Lambrianides, Turner-Stokes, Pericleous, Papadimitraki, Ioannou, Lawrie, Chen, Latchman, Rahman, Giles.
